# Validation of biomarkers of food intake—critical assessment of candidate biomarkers

**DOI:** 10.1186/s12263-018-0603-9

**Published:** 2018-05-30

**Authors:** L. O. Dragsted, Q. Gao, A. Scalbert, G. Vergères, M. Kolehmainen, C. Manach, L. Brennan, L. A. Afman, D. S. Wishart, C. Andres Lacueva, M. Garcia-Aloy, H. Verhagen, E. J. M. Feskens, G. Praticò

**Affiliations:** 1 Department of Nutrition, Exercise and Sports, University of Copenhagen, Copenhagen, Denmark; 2 Department of Food Science, University of Copenhagen, Copenhagen, Denmark; 3 International Agency for Research on Cancer (IARC), Nutrition and Metabolism Section, Biomarkers Group, Lyon, France; 4 Agroscope, Federal Office of Agriculture, Berne, Switzerland; 5 University of Eastern Finland, Kuopio, Finland; 6 INRA, Human Nutrition Unit, Université Clermont Auvergne, F63000 Clermont-Ferrand, France; 7 UCD Institute of Food and Health, UCD School of Agriculture and Food Science, University College Dublin, Dublin, Ireland; 8 Division of Human Nutrition, Wageningen University and Research, Wageningen, The Netherlands; 9 Department of Biological Sciences, University of Alberta, Edmonton, Canada; 10 Biomarkers and Nutrimetabolomics Laboratory, Department of Nutrition, Food Sciences and Gastronomy, University of Barcelona, Barcelona, Spain; 11 CIBER de Fragilidad y Envejecimiento Saludable (CIBERFES), Instituto de Salud Carlos III, Barcelona, Spain; 12 European Food Safety Authority (EFSA), Parma, Italy; 13 University of Ulster, Coleraine, NIR UK

**Keywords:** Biomarker, Validation, Nutrition, Assessment of food intake, Metabolomics, Review

## Abstract

Biomarkers of food intake (BFIs) are a promising tool for limiting misclassification in nutrition research where more subjective dietary assessment instruments are used. They may also be used to assess compliance to dietary guidelines or to a dietary intervention. Biomarkers therefore hold promise for direct and objective measurement of food intake. However, the number of comprehensively validated biomarkers of food intake is limited to just a few. Many new candidate biomarkers emerge from metabolic profiling studies and from advances in food chemistry. Furthermore, candidate food intake biomarkers may also be identified based on extensive literature reviews such as described in the guidelines for Biomarker of Food Intake Reviews (BFIRev). To systematically and critically assess the validity of candidate biomarkers of food intake, it is necessary to outline and streamline an optimal and reproducible validation process. A consensus-based procedure was used to provide and evaluate a set of the most important criteria for systematic validation of BFIs. As a result, a validation procedure was developed including eight criteria, plausibility, dose-response, time-response, robustness, reliability, stability, analytical performance, and inter-laboratory reproducibility. The validation has a dual purpose: (1) to estimate the current level of validation of candidate biomarkers of food intake based on an objective and systematic approach and (2) to pinpoint which additional studies are needed to provide full validation of each candidate biomarker of food intake. This position paper on biomarker of food intake validation outlines the second step of the BFIRev procedure but may also be used as such for validation of new candidate biomarkers identified, e.g., in food metabolomic studies.

## Background

Quantitative assessment of food intake is normally done by the use of questionnaires, diaries, or interviews [1, 2]. These instruments are error-prone due to their subjective nature [3]. The use of qualitative biomarkers to assess recent food intake could be a qualification tool to improve the value of current instruments for food intake assessment. Further development of such biomarkers of food intake to improve their use for quantitative assessment of recent or more long-term food intake could be a long-term goal in this field. In a recent paper, we have suggested a flexible classification scheme for biomarkers used in nutrition-related health research [4]. According to this scheme, the biomarker classification is determined by the intended use of the biomarker measurement. An important additional issue relates to biomarker validation. Such validation will also depend on the purpose of using the biomarker, i.e., on how the measurement may be interpreted. The development of a validation scheme would therefore be necessary for each of the different biomarker classes. Within the FoodBAll consortium (www.foodmetabolome.org), the class of biomarkers of food intake (BFIs) is the main focus area. A large number of candidate BFIs are currently being observed in food metabolomic studies, and others are found by extensive literature reviews such as those following the BFIRev guidelines [5]. There are also intervention studies covering a range of foods performed in the FoodBAll project to discover new biomarkers of food intake. This gives hope that candidate biomarkers may be found for a large number of foods. However, they must be validated to assure that they accurately represent the level of intake of the food considered, that the sample type and time of sampling is appropriate for the intended use, and that the analytical method is valid according to current standards.

Validation of a BFI is not only a matter of analytical validity measured according to standards [6] but also a matter of biological (nutritional) validity, i.e., what it represents in terms of intake of a specific food under the conditions of the study. Clearly, this will depend on factors such as variability of the content of biomarker precursors in foods and of their metabolism and kinetics in individuals. Therefore, validation criteria must include also biological aspects of the biomarker [7]. It is also important that the validity of a BFI is reconsidered for the intended purpose whenever it is applied. Some efforts have been made previously to evaluate biomarkers for food or dietary intake in nutrition studies. For instance, de Vries et al. proposed criteria to assess the meaningfulness of surrogate markers and pointed out that no clear criteria could be set up for dietary intake markers, dietary exposure markers, and nutritional status markers due to the lack of established validation criteria [8]; Scalbert et al. suggested a grading system for BFIs to indicate their level of validation. This approach introduces the concept of stepwise improvement of validity, e.g., by the use of a score or a set of criteria [9]. Several authors have pointed out that a number of factors such as kinetic variables, factors related to sampling and storage, and factors related to the variability of food composition are important determinants of measurement error beyond simple analytical error [10–14]. However, there is no systematic method established to validate BFIs. Here, we propose a validation scheme for this particular group of biomarkers. For newly discovered candidate BFIs, the suggested validation criteria incorporate analytical and biological aspects into a common system using eight aspects of validation to allow partial or full validation. Our purpose was to review the current stage of BFI validation and to suggest appropriate steps for assessing the validity of candidate BFIs. By applying this validation scheme, researchers could obtain the information needed to make good use of the BFIs and an overview of the additional studies needed for the development of the BFIs. To our knowledge, this is the first comprehensive scheme developed for this area. The resulting validation criteria and methodology have been tested using *Allium* intake biomarkers as an example and will subsequently be applied on candidate BFIs for all major foods or food groups. These reviews will be published in this thematic issue of *Genes & Nutrition*.

## Methods

**Fig. 1:**
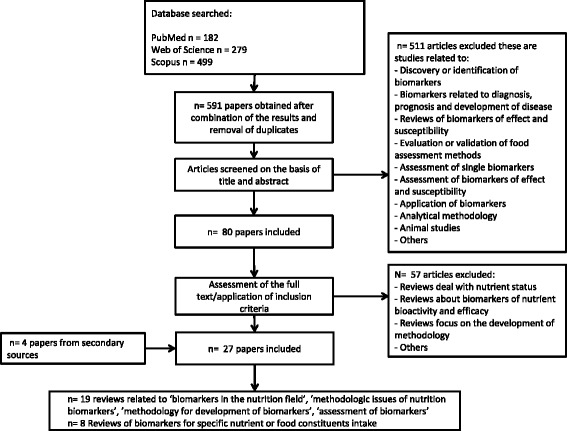
Flow diagram of search for papers on criteria for biomarker validation in nutrition field

In order to identify papers dealing with the validation of BFIs, we carried out an extensive literature search following the BFIRev methodology proposed previously [4]. Briefly, searches were carried out in three databases (PubMed, Scopus, and ISI Web of Knowledge) in November 2016. In PubMed, the search terms were (nutrition*[Title/Abstract]) AND (biomarker*[Title] OR marker*[Title]) AND (validation*[Title/Abstract] OR validity*[Title/Abstract] OR validate*[Title/Abstract] OR assessment*[Title/Abstract]) NOT (animal OR rat OR mouse OR mice OR pig) NOT (disease*[Title] OR risk*[Title] OR inflammat*[Title/Abstract] OR patient*[Title]). To avoid all the studies concerned with a single biomarker while keeping studies on validation in general, we avoided using nutrient* or food* in the search strategy. The fields used for the other two databases were [Article Title/Abstract/Keywords] for Scopus and [Topic] for ISI Web of Science to replace [Title/Abstract] for PubMed. The search was limited to papers in English language and with no restriction applied for the publication dates. The review papers discussing the development and application of biomarkers in the nutrition field were selected in the process outlined in Fig. 1. The first draft scheme of validation criteria was based on criteria proposed in the review papers found by this literature search. This list was revised by three rounds of commenting by co-authors as well as feedback from presentations at international conferences.

## Results

The literature search provided a range of factors to be considered for the validation and application of biomarkers in general or for BFIs in particular. These were sub-grouped to provide eight separate characteristics that may together comprise biomarker validity for BFIs (Table 1). There is no intended hierarchy in the order.

**Table 1:** Factors to be considered for the validation and application of biomarkers suggested in previous literature

Characteristic	Factors to be considered for the validation and application of biomarkers	References
1. Plausibility	• Biomarkers should be specific to the food (having the ability to distinguish the food or food component of interest from other foods or food components). • There should be a food chemistry or experimentally based explanation for why the food intake should increase the biomarker, e.g., the biomarker should be a metabolite of a food component.	[71–81]
2. Dose-response	• Evaluation of the dose-response relationship should be performed to assess the suitability of the biomarker over a range of intakes. • Limit of detection should be evaluated to provide the information about how responsive (sensitive) the biomarker is. • Baseline habitual level needs to be established. • Bioavailability of (the precursor of) the biomarker should be evaluated to provide the information about its sensitivity to intake. • Detailed information about saturation effects of the biomarker should be known.	[9, 10, 12, 13, 71, 73–88]
3. Time-response	• The half-life of the biomarker should be evaluated to specify the degree to which a biomarker reflects exposure, e.g., days, weeks, months or years. • Kinetics (comprises “formation, distribution, metabolism and excretion”) should be known to make choices, such as on sampling time and matrices. • Timing of measurement in relation to bioavailability and bioefficacy must be considered. • Temporal relation of the biomarker with dietary intake should be considered to provide information for choosing types of specimen. • Repeated measures of the biomarker over time should be evaluated to provide insight into the reproducibility of biomarker concentrations, and thus, the likelihood that the biomarker is a stable estimate of long-term intake.	[9, 10, 12, 71, 74, 76, 77, 79, 82, 85–87, 89]
4. Robustness	• Suitability of the biomarker in a free-living population should be investigated using a (controlled) habitual diet to provide information such as its interactions with other foods and its applicability to a certain group of population. • The biomarker should be validated in a controlled dietary intervention studies as well as in cross-sectional studies. • Validation of the biomarker in different subjects and study settings is needed. • Information such as interactions with other food components and influence of food matrix should be excluded or known to be manageable.	[12, 76, 80, 85]
5. Reliability	• Comparison of the biomarker and a gold standard or reference method that provides a good measure of the true exposure is necessary. • Biomarkers identified using samples from cohort studies should ideally be combined with intervention studies to demonstrate their direct relationships with intake. • Comparison between the biomarker and an appropriate dietary assessment method should be performed. • A biomarker should be confirmed in accordance with other biomarkers for the same food or foods. • Validation of a biomarker can be attempted by measuring it in animals fed different nutrient intake under tightly controlled conditions.	[9, 11, 13, 71, 72, 74, 75, 77, 84, 87, 90]
6. Stability	• Suitable protocols for sample collection, processing, and storage are needed to keep the sample quality for several years. • Trials should be carried out to determine whether analytes undergo decomposition during storage.	[76]
7. Analytical performance	• Precision, accuracy, and detection limits of the method should be evaluated. • Comparison against validated methodology or references or references materials is needed. • The calculation of inter- and intra-batch variation should be performed. • Statistical quality control procedures (coefficient of variance, standard deviation and inaccuracy limits for data) should be established.	[10–13, 71–74, 76, 82, 84, 91]
8. Reproducibility	• There is the need to develop and use accurate and validated analytical methods to adequately compare the data obtained in different laboratories.	[12, 71, 78]

A validation assessment system (questions 1–8) was developed based on these characteristics. Validation can be assessed for each candidate biomarker by evaluating the current evidence related to each of the characteristics proposed, thereby answering the related question. Possible answers are Y (yes, the criterion is fulfilled for at least some use of the biomarker), N (the criterion has been investigated but it was not fulfilled), or U (the criterion has not been investigated or data is not available). Following the commenting rounds, the eight aspects of validation remained although some of them are clearly complex and may therefore only be answered positively under certain conditions and these conditions must therefore be stated. The validation questions and their sub-criteria for answering “yes” are shown in Table 2.

**Table 2:** Eight groups of validity criteria for biomarkers of food intake

Validation criterion	Validation questions and their sub-criteria for answering “yes”	
1. Plausibility	*Q: Is the marker compound plausible as a specific BFI for the food or food group (chemical/biological plausibility)?* ((The BFI is likely to be a metabolite or process-related derivative of a compound known to occur in the food or food group) OR (the BFI has been identified as a putative biomarker for the food/food group) OR (the compound was identified as a putative biomarker in a metabolomics investigation)) AND (variability of (parent) compound within food or food group (if known) is limited) AND ((the level of the (parent) compound in other foods is comparatively low) OR (presence only in other foods not commonly consumed))	
2. Dose-response	*Q: Is there a dose-response relationship at relevant intake levels of the targeted food (quantitative aspect)?* (The dose-response relationship of the BFI has been established using several intake levels (in a meal study) OR (in different meal studies where the results were comparable) OR (in cross-sectional study or longitudinal observational studies)) AND (the background level of the BFI is 0 or low) Information about the limits for common background levels and saturation kinetics of the BFI should be provided as a comment	
3. Time-response	*Q: Is the biomarker kinetics described adequately to make a wise choice of sample type, frequency and time window (time-response)?* a. (The single-meal time-response relationship of the BFI has been described for a defined sample type and time window in a meal study) OR	
	b. (The kinetics of the BFI after repeated intakes has been described for a defined sample type in a meal study) OR (accumulation of the BFI in certain sample types has been observed) Information about ADME and enzyme induction, inhibition, or altered excretion in the metabolism of the BFI or its precursor could be provided as a comment.	
4. Robustness	*Q: Has the marker been shown to be robust after intake of complex meals reflecting dietary habits of the targeted population (robustness)?* ((The BFI has been measured and shown to be robust after intake of complex meals (in intervention studies) OR (in observational studies)) AND ((There is no confounding food observed) OR (The level of the BFI from the confounding food is low) OR (The confounding foods are not commonly consumed))	
5. Reliability	*Q: Has the marker been shown to compare well with other markers or questionnaire data for the same food/food group (reliability)?* (The BFI has been compared well (with other biomarkers for the same food or food group) OR (with dietary assessment instruments) OR (with data in studies with highly controlled setting and supervised intake)	
6. Stability	*Q: Is the marker chemically and biologically stable during biospecimen collection and storage, making measurements reliable and feasible (stability)?* (The BFI is chemically and biologically stable during biospecimen collection, processing and storage) OR (The BFI is not stable but suitable protocol has been established to achieve the stabilisation of the BFI)	
7. Analytical performance	*Q: Are analytical variability (CV%), accuracy, sensitivity and specificity known as adequate for at least one reported analytical method (analytical performance)?* (The protocol of the method has been well described and could be repeated) AND (The method has been compared with validated method or references) AND (The analytical variability (CV%), accuracy, sensitivity and specificity have been described)	
8. Reproducibility	*Q: Has the analysis been successfully reproduced in another laboratory (reproducibility)?* (The analysis with the same method has been performed in at least 2 different laboratories) AND (The measurements of the BFI obtained from different laboratories are comparable)	

The first five criteria are related to the biological validity and applicability in nutrition research; the remaining three criteria are related to their analytical performance. Many of these aspects may depend on the intended application of the BFI, and the validity cannot therefore be answered unambiguously in all cases. The criteria should be seen as representative of the most important aspects of the validation to be considered in the systematic evaluation of each BFI. The conditions under which the BFI is valid must therefore be added to qualify the validation. In their most simple form, the validation criteria would translate into the questions added in the table.

Assessment of validity should consequently be possible to perform in a reliable and documented manner using these questions. For Y and N answers, the conditions under which the validity has been assessed may be stated as a comment. For instance, trimethylamine oxide (TMAO) may be a valid short-term biomarker for intake of cold-water fish from the sea when measured in urine samples [15]. However, contents of TMAO may be lower in some other fish making the marker unreliable for certain geographical areas [16].

The validation criteria may also be seen as criteria for prioritization of further work on biomarker validation. Whenever a question is answered by “N” or “U,” there is a need for an additional work to provide the lacking information. However, a conditional “Y” would also indicate that further work may be needed. This should help to identify the most promising BFIs and to plan any validation work still needed in order to fully validate them. The system is not geared towards scoring of validity since each criterion may have different weight in different applications of a biomarker. So validity may be sufficient without eight Ys for some use of a biomarker. For instance, the short-term kinetics would normally be irrelevant for biomarkers to be measured in hair samples where only multiple-dose kinetics is of importance.

## Discussion

Based on literature review and a consensus procedure, we divided aspects of biological (nutritional) and analytical validity into eight criteria. The underlying aspects of each criterion may include several potential sources of unintended variability in food intake assessments using the BFI in question. While a similar procedure has been suggested previously [9], this is to our knowledge the first attempt to comprehensively and systematically develop a validation procedure for BFIs. When applying this validation procedure, it is important to be specific about the intended use. This could be assessment of intake of a specific food or the whole food group. For instance, biofluid measurements of caffeine may be seen as a BFI for the food group of caffeinated foods and drinks while being less useful for any specific drink such as coffee. It may also be reasonable to include aspects of variability such as that caused by metabolism. Caffeine measurement would reveal recent intake of caffeine-containing products, but it is extensively metabolized. Caffeine metabolism has variable kinetics based on common variation in genetics and lifestyle factors [17, 18], and this might cause limitations for quantitative use. It could therefore be considered whether the sum of major caffeine metabolites in a 24-h urine might perform better as a quantitative BFI for this class of foods [19].

Candidate BFIs have often been based mainly on information from food chemistry pointing to special compounds found only in specific foods or food groups. The relatively well-studied alkylresorcinols BFIs of (whole grain) wheat and rye intake are a good example of this [20, 21]. More recently, BFIs are frequently suggested from metabolomic studies. For instance, proline betaine was observed in several early studies and further confirmed as a BFI for citrus by Heinzmann et al. [22]. The metabolic profiling studies can have highly variable designs, e.g., experimental meal studies, dietary intervention studies, or cross-sectional studies [9, 23]. The study in which a BFI is initially discovered may additionally provide some information related to one or more criteria of validation. This depends on the study design, e.g., plausibility based on food chemistry studies, kinetics based on postprandial concentrations in plasma or urine from meal studies, quantitative aspect based on different levels of exposures in dietary interventions, or robustness based on findings from a cross-sectional or other observational setting.

Each of the eight criteria defined above is further discussed below along with suggestions on the studies and sample methods most suitable for validation. Since none of the validation questions in Table 2 may be answered without influence of some of the aspects covered by one or more other criteria, the discussion of each criterion also includes some of the interrelations with the others.

1. Plausibility. The amount of analytical information on foods and food groups is extensive and increasing rapidly with the use of metabolomics in food chemistry. Available knowledge on food composition and food compound metabolism is more and more integrated into online databases (FooDB, Phenol-Explorer, PhytoHub, etc.), which facilitate the identification of biomarkers specific for individual foods. A candidate BFI may therefore be suggested and evaluated based on the food chemistry literature as already exemplified in the case of the alkylresorcinols. An important aspect here is the variability of contents within the food source, e.g., as a result of different varieties, and growth conditions. High and unpredictable variability may reduce the usefulness of a food compound as a BFI; this may for instance be the case for feed and pasture-derived compounds in dairy products [24]. Another possible origin of a candidate BFI could be the food processing such as fermentation or heating. For instance, during the cooking of meat, heterocyclic aromatic amines could be formed from creatinine, creatine, and amino acids at high temperature, which may be a biomarker of intensively cooked meat intake [25]. As an example of more complex markers based on food production, four different beer metabolites have been proposed as a combined marker of beer intake, two of them reflecting beer raw materials (hops and barley) and the other two reflecting the production processes (malting and fermentation), respectively [26]. If a candidate biomarker is highly specific (only minor interference is expected from other food sources), or unique for the food or food group in question, it is likely to have good plausibility. Interference from other sources may be expected to be low because other potential sources have either low content or a very low level of consumption in the population considered. For newly suggested BFIs, this check based on current knowledge from food chemistry in combination with knowledge on possible host metabolism may represent the first step to evaluate chemical and biological plausibility. Plausibility is an essential criterion for any BFI but not sufficient, as a very specific compound may for example be too variable in contents in the food, too unstable in the food or in body fluids, or be unreliable due to a high inter-individual variation in ADME; some of these aspects are covered below.
2. Dose-response after a single exposure. This validation criterion may be satisfied if short-term or long-term dose-response relationship in humans has been clearly established for the candidate biomarker. For compounds with short half-lives, this may be accomplished with meal studies using several intake levels of a single food having a known content of the BFI or its precursor. Short-term dose-response information may also be achieved by analyzing cross-sectional or longitudinal data where 24 h records of food intake is available together with appropriate biological samples. BFIs with longer half-lives may not show postprandial dose-response kinetics at all and still be good biomarkers for assessing longer term ingestion of a food. Lipid-soluble compounds such as lycopene are examples of this [27]. For compounds like carotenoids, which are transferred into deeper compartments and only slowly released it is not possible to assess their short-term kinetics in blood after a single exposure [28]. Postprandial dose-response is therefore obviously not always needed for validation and must therefore often be answered together with a comment explaining the possible reason for the lack of dose-response. The presence of postprandial dose-response indicates that the marker has relatively fast kinetics into the body fluid sampled and that background levels are low compared to the change following food intake. This would happen for relatively water soluble, uniquely food-derived compounds that diffuse or are transported into the blood, that are not removed by the liver in the first pass, that are not the subject of multiple pathways of degradation, and that are excreted at a similar rate in most subjects. A typical example of such a BFI is proline betaine, which is almost inert to human metabolism while reflecting recent exposure to citrus fruit products [22]. For the measurement of urinary 1-methylhistidine, representing meat intake, postprandial dose-response is visible in highly controlled studies but the background level is too high so that lower meat intakes cannot be detected [29–31]. Excretion of 3-methylhistidine is therefore preferred, but this compound is not found in all meats and seems to be a better marker for chicken than for others [31]. Actually, the situation for 1-methylhistidine is not unusual and many biomarkers may have background levels due to low levels in some other foods and/or to endogenous formation. Even proline betaine is present at 100–1000 times lower levels in other fruits and vegetables, so a low background level may be seen even without citrus exposure [22]. Whenever possible, the limits for common background levels may therefore be added in a comment to this criterion to describe levels that do not indicate exposure to the target food. Any indication of saturation kinetics at higher intake levels may be another phenomenon that may be relevant to note in BFI short-term dose-response studies although saturation phenomena in nutrition are currently best known from nutrient intake biomarkers (NIBs) such as ascorbate.
3. a. Time-response after a single exposure. This question relates to the optimal time window for measurement of a BFI. This depends on the uptake or elimination half-life of the BFI determined after a single exposure to the food. The importance of this criterion may again depend on the intended application; it is important in order to point out whether there is evidence to use the BFI for a defined sample type and time window. The factors causing variability in postprandial dose-response (variable contents in foods, variable ADME) would also apply here. Qualifying statements relating to food and individual variability may consequently be needed in addition to Y or N. Absorption that leads to measurable levels of the BFI in blood over an extended number of hours would mean that blood samples may be useful for food intake assessment within that specified time interval after food intake. BFIs with fast metabolism or excretion would narrow the useful time window for blood samples. Proline betaine showing fast absorption and excretion may be measured in blood in only a short time interval, whereas collection of urine over a time span of a few hours or more would recover almost all proline betaine ingested in that time interval. Urine samples would therefore represent recent intakes before or during biospecimen collection for these BFIs. This is important because repeated urine sampling over a time period may be used to represent the frequency of intake of the target food in the study period or in the habitual diet of the study subjects [25]. The urine sampling plan (frequency and duration of collection) will therefore determine the ability of a study to provide food intake information for BFIs with shorter half-lives. Late absorption and excretion occurs for BFIs that depend on release by the gut microbiota. An example of such markers is the urolithins which are formed by certain microbes during degradation of ellagic acid from berries and nuts [32, 33]. These markers are only excreted after 24–36 h following intake of ellagitannins and may peak at 48–72 h [34, 35]. For BFIs with complex absorption kinetics and/or a very long elimination half-life after absorption such as the carotenoids, the single-dose exposure kinetics may be of less relevance because background levels are usually high. Repeated-dose kinetics (criterion 3b) is more important for these BFIs
  b. Time-response after multiple exposures. The time-response after single exposures may need additional considerations related to repeated intakes of the same food or food group. The time-response after multiple exposures includes phenomena such as accumulation in hair or blood plasma or cumulative increases in excretion. This information is of importance to select the best possible sample type and timing of the sample collection for the assessment of habitual intake. Accumulation of the biomarker in blood, hair, or nails is affected by repeated exposures to some foods, and therefore, it could reflect the current habitual intakes. For instance, the measurement of alcoholic beverage intake by ethyl glucuronide in hair may be suitable for estimating habitual intakes since it builds up in hair after exposure to multiple doses in the longer term [36]. This would not be the case for urine or blood where the presence of the marker reflects only recent intake. However, further characterization of this marker for different kinds of hair, for subjects with different polymorphisms of ethanol metabolism, etc. may still be needed in order to fully validate this marker for quantitative longer term intake assessment [37]. Most BFIs have not been studied in hair and more studies are needed to evaluate the usefulness of hair or nail clippings for BFIs currently measured mainly in blood or urine. For the medium-range or slowly excreted food-derived compounds such as lycopene, quercetin, or lipids, plasma levels may build up. For instance, after consumption of tomato, the plasma level of lycopene needs 3–4 days to return to baseline, which makes it a good biomarker for habitual tomato intake in most cases where the frequency of tomato ingestion is more than once a week [24]. For quercetin with an excretion half-life of around 16–20 h, this would still allow plasma kinetics to be studied after a single meal in most cases, whereas plasma levels of C22:6 fatty acids from seafood may not change appreciably after a single seafood meal in habitual consumers of fatty fish but only as a consequence of repeated exposures making blood samples potentially useful for measuring habitual intakes [38, 39]. The accumulation as a consequence of different dietary levels should therefore be evaluated for this BFI. Enzyme induction, inhibition, or altered excretion may affect kinetics of elimination after repeated exposures. Foods such as coffee, garlic, and cabbages contain inducers of phase 1 or phase 2 metabolism, including diterpenes, disulphides, indoles, and isothiocyanates. These phenomena are not well studied in humans while animal studies indicate efficacy of these compounds in enzyme induction [40]. When induction may be expected, it should be evaluated whether it might affect the use of the BFI, in particular whether this effect may dominate over other sources of variability. Other commonly ingested foods might also influence the ADME of a candidate BFI so that its kinetics may depend on the food matrix or even culinary culture as shortly discussed below under criterion 4.
4. Robustness in studies with complex diets. It is important to evaluate the robustness of the BFI when it is intended for use in observational studies with complex meals or diets. Many candidate BFIs have been suggested based on a limited number of intervention studies with highly controlled diets or on food chemistry knowledge. However, these data may not be sufficient to identify all other possible dietary sources of the BFI. For instance, limonene metabolites may be observed as good candidate markers of citrus intake in a controlled intervention study. Since limonene is also very abundant in citrus flavored foods (sweets, cakes, etc.), the use of it as a BFI would potentially lead to wrong conclusions counting unhealthy foods as fruit [41]. Whereas criterion 1, plausibility, is based on food chemistry literature, robustness is evaluated based on actual proof of the uniqueness of the BFI under conditions where multiple other foods are consumed at the same time. Such studies are typically cross-sectional or prospective studies where intake is monitored by food diaries or dietary recalls. For example, robustness of proline betaine was confirmed as it appeared as a biomarker predicting citrus intake versus no intake independently of study design; this included a cross-sectional study where citrus fruit intake was monitored by 24 h records in a free-living population, a fully controlled meal study with citrus, and a 4-week intervention with orange juice [42]. In a few cases, robustness may also be judged based on multiple complex meals containing one of the foods of interest [43]. Applicability of a BFI in populations with different food cultures or production systems may require an examination of the BFI robustness in each population. For instance, δ^13^C has been suggested as a BFI of added sugar refined from C4 plants such as corn, sugar cane, and sorghum. It works well in a population whose major source of sugar is C4 plants such as subjects in Mexico, Canada, and USA. However, for Europeans or Japanese who largely rely on sugar beet, a C3 plant, the use of δ^13^C may underestimate the intake of added sugar [44, 45]. Another aspect of robustness is the influence of other foods on the metabolism and kinetics of the BFI. This aspect is not well studied but may be indicated from some observations. For instance, the disruption of fat micelle formation in the gut by foods rich in plant sterols leading to reduced cholesterol uptake [46] may also affect other lipid-soluble compounds, but so far, this has only been shown to affect carotenoids [47]. So in a comparison of subjects with differences in habitual plant sterol intake, carotenoids may in theory not estimate intake of plant foods in a balanced way. However, direct evidence for quantitative importance is still lacking.
5. Reliability based on other markers of intake for the food in question. Reliability is traditionally the comparison of a new biomarker against the current best (gold) standard methodology [13]. This validation of a BFI should ideally be done in a highly controlled setting with supervised intake so that the exact amount of the food consumed is monitored for each volunteer in the study. In such a study, direct comparison by plots such as Bland-Altman and/or Passing-Bablok can be performed for exact validation and outlier detection [48, 49]. Alternatively, the new BFI is validated against a previously validated “gold standard” for intake assessment of the food in question, but such a method is only rarely available for BFIs. So the current best practice may be the use of dietary assessment instruments. Validated questionnaire data, food records, or diaries may be available to judge the reliability of the marker. This is not ideal since it implies validation of a potentially more objective and precise instrument by less precise and subjective information. Depending on the precision of the dietary instrument, the quality of the validation by this criterion will vary. Food diaries and 24-h dietary recalls covering the day of blood or urine collections for BFI measurement should be preferred over food frequency questionnaires for reliability assessments. A useful resource is the Exposome-Explorer database in which over 8000 correlation values between biomarker levels and intake of a large diversity of foods have been curated for a large number of BFIs [50]. In some cases, several new candidate markers are found simultaneously by metabolomics [51]. Such new BFIs representing the same food may be validated for reliability against each other in a separate analysis to compare their capacity to accurately predict food intake [52]. This latter strategy is not without pitfalls as exemplified above with proline betaine and limonene metabolites, both markers of citrus intake but with vastly different robustness in mixed meal studies [41]. This strategy for evaluating reliability should therefore be interpreted with care, preferably using data from different study designs; observational evidence of reliability may for instance be confirmed in a controlled trial or evidence from controlled studies may find confirmation from a cross-sectional setting where several different intake levels can provide information about concordance between the candidate BFIs. Simultaneous use of information from dietary assessment instruments in such studies as described above would help to assure that the markers also agree reasonably with subjective food intake data.
6. Stability of the BFI. This validation criterion is related to best practices for sample collection and storage. Compound structure, sample collection, storage and handling, and sample pre-processing should be considered when evaluating the stability aspect for an intended use of a candidate biomarker. Many metabolites have been found to be quite stable over time under conditions of low-temperature storage [53]. However, both temperature and environment are important determinants. It is generally accepted that storage at temperatures of − 20 °C or higher is suboptimal and leads to oxidative degradation. However, systematic studies of storage stability at − 80 °C over a longer time period are very few beyond a few years [54] and have not covered many food compounds, so the practice of sample storage for 5–10 years used for many cohorts and experimental studies is not well documented. Storage stability in these cases may be evaluated by comparing the distribution of concentrations measured in a set of stored and fresh samples of comparable origin or by repeated analyses of a set of QC samples, stored in multiplicates. Storage at even lower temperature and under a nitrogen atmosphere is probably ideal and even enzyme activities seem to be in the normal range after more than 10 years of storage under such conditions [55, 56]. Inherent compound stability and potential for enzymatic breakdown during sampling is another issue that must be carefully considered under this validation criterion. For instance, highly oxidizable compounds such as beer humulones may degrade during storage of the beverage as well as during collection of urine samples voided into an oxygen-containing collection jar. Such compounds may only serve as BFIs when both the parent compound and the products are known and measured [26]. Other potential degradation pathways include pH instability and metabolism by enzymes or cells present in the preparation. Special collection conditions may be needed for stabilizing certain BFIs, e.g., special tubes for ascorbate and glucose stabilization as well as urine collection at pH below 2 for anthocyanins [57–59].
7. Analytical performance of the BFI measurement. Reliable chemical analysis is of central importance for any BFI. Several analytical quality aspects exist, including precision, accuracy, and intra-batch and inter-batch variability; however, these are concatenated here into a single validation criterion to assess whether qualitative or quantitative analysis of the BFI is feasible. Comments to Y answers are therefore mandatory for this criterion in order to qualify the statement by providing details of the analytical performance of the BFI analysis method. Few BFIs have been thoroughly validated in targeted analytical procedures by modern standards of analytical quality and what is sufficient may depend heavily on the intended use, e.g., qualitative or quantitative use. A candidate biomarker found by metabolomics must obviously have been measured at different levels in a body fluid under the conditions of the untargeted analysis applied. This indicates potential for qualitative (or so-called semi-quantitative) use and reflects the minimal requirement for Y with the comment, “qualitative analysis only.” In studies where the BFI additionally reflected known graded differences in exposures, the development of the method applied into a targeted, quantitative analysis may be judged as feasible under similar experimental conditions. This would reflect the minimal evidence to indicate potential for development of a quantitative analytical method for the BFI, and this information may be added as a comment but quantitative use would still be uncertain until an analytically validated method has been developed. The adequacy of an intended analytical method should therefore be carefully considered before application of a BFI with this kind of minimal evidence for analytical performance. In most instances, a thorough analytical method validation for a biomarker is not made until a dedicated targeted method is being developed. This question should therefore be answered positively only with an accompanying comment on its potential for use in qualitative and quantitative applications. For the former, a sufficient limit of detection may be adequate. For the latter, a targeted method which has been analytically validated according to current recommendations by analytical chemistry journals or societies [60–62] is needed to assure uncompromised use of the BFI. For full validation, it is necessary to use an isotope-labelled standard for the BFI as reference in every sample, but such standards are not available for most compounds. However, new methods for derivatization with labelled agents may help solve this issue, depending on the compound structure [63–65]. For instance, free short-chain fatty acids may be measured quantitatively using both a labelled and an unlabelled agent for derivatization of carboxylic acids [66].
8. Reproducibility across laboratories. Measurement of a BFI should give the same result when analyzed in different laboratories. Repeatability is indicated when the same analysis of the marker has been reproduced in at least two different laboratories but should eventually be evaluated by inter-laboratory comparison tests. Such tests apply the final, targeted analysis of the BFI in a common set of samples distributed in a blinded fashion to the participating laboratories. Inter-laboratory comparisons are often used for assessment of laboratory performance [67], and this must also be considered if the procedure is used for validation; if one of the laboratories does not follow the analytical procedure well, the outcome may erroneously indicate that the BFI is not repeatable across laboratories. It is therefore preferable that several laboratories contribute. Inter-laboratory comparisons may even be performed with metabolomic methodology, i.e., before a fully validated analytical procedure has been developed [68, 69]; however, a carefully standardized metabolomics procedure should then be used by all participating laboratories to avoid misinterpretation of biomarker validity.

Variable levels of a BFI at a fixed food intake could come from differences in metabolism due to the age, sex, smoking, medicine; from influence of other dietary factors or microbiota; from factors affecting stability of the marker; or from variable contents of the biomarker precursor in the food. Variability is also seen within an individual due to several of these factors. Biomarker variability is an important issue across most criteria but has not been considered a criterion as such because many aspects of variation can be controlled technically by careful sampling, analytical procedures, and statistical handling. The un-controllable factors are the individual differences in metabolism, variable contents in the food, and food matrix effects. When they are large compared to the variation in intakes of the food in question, the biomarker may not be useful and this will be observed in careful dose-response and time-response studies (criteria 2 and 3) and in studies of robustness (criterion 4). For instance, the sulphate conjugate of 4-ethyl-5-methylamino-pyrocatechol was observed having an apparent dose-response relationship with beetroot intake in a parallel intervention study [43]. The dose-response curve clearly indicated considerable variability; such variability may be due to variation in the presence of the parent betalain in the beetroot dishes consumed or to differences between individuals in betalain metabolism. This could be caused by large inter-individual variations in its endogenous metabolism (hydrolysis and conjugation) and in metabolism by the microbiota. Extensive metabolism or degradation could therefore constitute a drawback for the use of this BFI considering that its variation is high compared with the variations in intake. For microbial metabolites in general, their presence may depend on the presence of a certain metabolic functionality of the microbiota. As a consequence, they may show major variation between individuals, making them less useful as BFIs, as demonstrated for the urolithins [70]. Finally, apparent variation in sample concentrations at a certain food intake may be caused by the use of food diaries as reference measurement since volunteers may not correctly note the ingested amount of the food in question. Controlled dietary studies are therefore needed to investigate variability. Most of these sources of variability are not only affecting the validation process but also affect the interpretation of validated food intake biomarkers. Careful and repeated sampling and/or use of markers with longer half-lives tend to reduce the influence of variable contents in the food or intake levels. Variable metabolism is more difficult to control by technical means and could render a BFI useful only at the group or population level.

By these eight validation questions, the current status of biological/analytical validation, including reliability and robustness of a biomarker, can be assessed. For the purpose of reviews on BFIs including their validation according to the current criteria, the number of questions answered “Y” may be used as a score; however, since the questions may not be equally important for all BFIs, the application of such a score to rank BFIs according to validity may be misleading. More rigid criteria for when to answer Y or N to each of the criteria could be helpful; however, the number of different scenarios to consider is very large and further work will be needed to delineate stricter criteria. The current validation approach is therefore based on explanatory comments to supplement the evaluation of Y or N. Although the validation is intended to provide a more authoritative guidance regarding the potential of a candidate biomarker, validity of a BFI will depend on the intended application and must always be considered by the user. The explanatory comments are therefore important for the end user to judge a given application.

When the validation criteria are applied for use of BFIs as qualitative markers, the scheme may be followed less stringently (Table 3). For instance, dose-response characteristics and analytical validation do not need to be documented in detail for qualitative BFIs since an all-or-none response is all that would be required. The presence of ethyl glucuronide in a blood sample, for instance, would clearly indicate that an alcoholic beverage has been ingested within the last 24 h. This measurement would suffice for assessment of compliance even if the analytical procedure is not done with internal standards. For this biomarker, the use of an internal standard would provide an accurate concentration and for a 24 h urine sample also the amount of beverage ingested recently. The number of criteria met as such may not be very informative, except for a rough estimate of how much further validation may be needed. For example, a biomarker having five Ys but with N for questions 1, 4, and 5 may not be useful at all. However, in case the lacking evidence is for questions 1 (parent food compound still unknown), 3b, and 8, it would seem quite reliable even for quantitative use since only inter-laboratory comparisons may additionally be needed. So as already underlined repeatedly, the user must still take care to check that any of the validation criteria may apply for the intended use and appreciate that overall validation can only be made for a defined application.

**Table 3:** Criteria need to be fulfilled for different uses of BFIs

Criterion	Experimental study (compliance biomarker)		Observational study	
	Qualitative	Quantitative	Qualitative	Quantitative
1 Plausibility	√	√	√	√
2 Dose-response		√		√
3a Time-response (single dose)	√	√	√	√
3b Time-response (multiple doses)		√	√	√
4 Robustness			√	√
5 Reliability	√	√	√	√
6 Stability	√	√	√	√
7 Analytical performance		√		√
8 Reproducibility		√		√

## Conclusions

This paper outlines a simple validation system for candidate BFIs identified from a literature search, from metabolomic studies, or from food chemistry. The validation criteria were identified from the literature and further grouped by the authors. The validation system has the advantage of pointing out the specific areas where a BFI is sufficiently validated while also highlighting those aspects where additional studies would be needed in order to provide improved validation. An important strength of this approach is therefore that it provides a stepwise strategy to improve the validity of existing BFIs as well as a test strategy for new candidate BFIs emerging from metabolomic studies, literature review, or from food chemistry. The validation system for BFIs proposed here includes aspects of plausibility, precision, stability, single or repeated intake kinetics, reliability, robustness, repeatability, comparability, and analytical performance. These criteria include all aspects of validation suggested in previous reviews on this topic. Although some of the criteria may simply be answered Y or N, commenting on specific conditions for the judgement of validity of a BFI may often be needed in order to pinpoint limitations on its use.
